# Identification of candidate genome regions controlling disease resistance in *Arachis*

**DOI:** 10.1186/1471-2229-9-112

**Published:** 2009-08-22

**Authors:** Soraya CM Leal-Bertioli, Ana Carolina VF José, Dione MT Alves-Freitas, Márcio C Moretzsohn, Patrícia M Guimarães, Stephan Nielen, Bruna S Vidigal, Rinaldo W Pereira, Jodie Pike, Alessandra P Fávero, Martin Parniske, Rajeev K Varshney, David J Bertioli

**Affiliations:** 1 Embrapa Genetic Resources and Biotechnology, C.P. 02372, CEP 70.770-900, Brasília, DF, Brazil; 2 Catholic University of Brasília, Campus II, SGAN 916, CEP 70.790-160, Brasília, DF, Brazil; 3 The Sainsbury Laboratory, Colney Lane, Norwich NR4 7UH, UK; 4 University of Munich Ludwig-Maximilians (LMU) Department of Biology, Maria-Ward-Strasse 1a, 80638, Munich, Germany; 5 International Crops Research Institute for the Semi-Arid Tropics (ICRISAT), Patancheru, Greater Hyderabad 502 324, India; 6 University of Brasília, Campus Darcy Ribeiro, Brasília, DF, Brazil

## Abstract

**Background:**

Worldwide, diseases are important reducers of peanut (*Arachis hypogaea*) yield. Sources of resistance against many diseases are available in cultivated peanut genotypes, although often not in farmer preferred varieties. Wild species generally harbor greater levels of resistance and even apparent immunity, although the linkage of agronomically un-adapted wild alleles with wild disease resistance genes is inevitable. Marker-assisted selection has the potential to facilitate the combination of both cultivated and wild resistance loci with agronomically adapted alleles. However, in peanut there is an almost complete lack of knowledge of the regions of the *Arachis *genome that control disease resistance.

**Results:**

In this work we identified candidate genome regions that control disease resistance. For this we placed candidate disease resistance genes and QTLs against late leaf spot disease on the genetic map of the A-genome of *Arachis*, which is based on microsatellite markers and legume anchor markers. These marker types are transferable within the genus *Arachis *and to other legumes respectively, enabling this map to be aligned to other *Arachis *maps and to maps of other legume crops including those with sequenced genomes. In total, 34 sequence-confirmed candidate disease resistance genes and five QTLs were mapped.

**Conclusion:**

Candidate genes and QTLs were distributed on all linkage groups except for the smallest, but the distribution was not even. Groupings of candidate genes and QTLs for late leaf spot resistance were apparent on the upper region of linkage group 4 and the lower region of linkage group 2, indicating that these regions are likely to control disease resistance.

## Background

The legume genus *Arachis *is of exclusively South American origin, and contains 80 described species [1,2]. By far the most economically important member of this genus is peanut, *Arachis hypogaea*. World annual production is about 35 million tonnes, more than 90% being grown by small farmers [3]. It is particularly important in Africa, where production greatly exceeds that of any other legume, and in Asia, where it provides more calories than soybean [4].

Diseases are important reducers of yield worldwide. Fungal foliar diseases of peanut such as rust (*Puccinia arachidis *Speg.), web blotch (*Phoma arachidicola *Marasas, Pauer, & Boerema) and early leaf spot (*Cercospora arachidicola *S. Hori) are important, but worldwide, late leaf spot (*Cercosporidium personatum *Berk. & M.A. Curtis) has the greatest impact. Sources of resistance against these and other diseases are available in cultivated peanut genotypes, although often not in farmer preferred varieties [5,6]. Wild species generally harbor greater levels of resistance and even apparent immunity, although the linkage of agronomically un-adapted wild alleles with wild disease resistance genes is inevitable.

In plant genomes disease resistance genes tend to occur in clusters. It seems likely that this distribution, which favors unequal crossing over and gene duplication and deletion, is an important factor in the evolution of the gene family and of new disease specificities [7,8]. Disease resistance gene clusters can comprise substantial portions of plant genomes, and understanding resistance gene clusters helps in the understanding of the structure and evolution of a plant genome as a whole. It also has implications for breeding because knowledge of the localization of resistance gene clusters would aid in the combination of disease resistances and alleles conferring desirable agronomic characters using marker-assisted selection (MAS) [9].

The map used in this work is based on a cross between the two A-genome species *A. duranensis *Krapov. & W.C. Gregory and *A. stenosperma *Krapov. & W.C. Gregory, the former being the most probable A-genome donor to cultivated peanut [10-12]. The aim of using these highly polymorphic wild diploids was to provide a reference map for peanut. This high level of polymorphism means a high percentage of candidate DNA markers are informative, thus facilitating the map's cross-referencing to other genetic maps. The map is based on microsatellite and legume anchor markers. Microsatellites were chosen because they are based on PCR, easy to use, and co-dominant. In addition they are highly transferable within the genus *Arachis *allowing the map to be integrated to other *Arachis *maps including the first recently constructed linkage map for cultivated peanut [13,14]. Legume anchor markers were chosen because they are transferable to other legumes and allow the alignment of this A-genome map with the maps of other crops and model legume species [15-18]. The A-genome map can thus serve as a "bridge" between, for instance, a low density map for cultivated peanut and the maps of other legumes, allowing information from different genetic maps to be accumulated.

In the present study we begin to define, on this map, the genomic regions that control disease resistance. For this, we placed candidate disease resistance genes and quantitative trait loci (QTLs) for resistance against late leaf spot on the map.

## Methods

### Mapping population

The mapping population of 93 F_2 _plants was derived from a cross between *A. duranensis *accession K7988 and *A. stenosperma *V10309, the same population used by [13]. DNA was extracted essentially as described by [19].

### Resistance gene analog marker development and genotyping

The nucleotide binding site domain (NBS) is found in numerous plant genes, and, to date has been exclusively associated with disease resistance. Therefore regions that encode NBS domains are excellent disease resistance gene candidates, and most of our focus was on homologs of genes encoding this domain, known as RGA (resistance gene analogs) markers. In addition, homologs of other genes known to be involved in defense resistance as well as genes that are induced upon challenge with pathogens [20] were mapped (full details and sequences of the mapped markers are in Additional file 1).

#### Southern blot

Nine clones representing all phylogenetic clades of NBS encoding regions described previously [21] were initially tested as probes for Southern hybridizations [22] with DNA from the parents and a limited number of F_2 _plants. Probes that showed higher polymorphism and easily scorable fragments were chosen for genotyping.

#### SCAR (sequence characterized amplified region) markers

Two primer pairs that amplify specific NBS encoding regions, both sequence confirmed [23], and one dominant SCAR marker derived from a bacterial artificial chromosome clone (Ad25F09-1; [24]) identified as containing a NBS encoding region by filter hybridization with clone S1_A_36 (Genbank ref. AY157808; [21]) were used for genotyping.

#### NBS profiling

For generation of markers derived from NBS encoding sequences we also used a modified AFLP (amplified fragment length polymorphism) technique known as NBS profiling. This was performed essentially as described by van der Linden and coworkers (2004) [25]. One hundred ng of genomic DNA was digested with *Pst*I and *Mse*I. Adapters were ligated to the restricted fragments using the following reaction mix: 500 nM *Pst*I adapter, 5 μM *Mse*I adapter, 1 mM ATP, 0.25 U/μl *Pst*I and *Mse*I restriction enzymes, 1 U/μl T4 DNA ligase and 1× the manufacturer's recommended reaction buffer, in a total volume of 10 μl. Fragments were pre-amplified by PCR with 300 nM of each primer P00 and M00, 200 nM of each dNTP, 0.5 mM MgCl_2_, 1× of manufacturer's supplied PCR buffer, 3.5 μl of template and 1 U of *Taq *DNA polymerase in a 50 μl volume. Dilutions (1:20) of these pre-amplifications were used as templates for amplification with primers designed for the kinase-2 motif of the NBS region of plant disease resistance genes, with varying levels of degeneracy, combined with primers designed to one of the adapters with varying numbers of selective bases (one, two or three). All primer sequences for NBS-profiling are described in Additional file 1. PCRs were performed with 1.5 μM of each primer, 1 μl of the ligation, 1× PCR buffer (as supplied by manufacturer of *Taq*), 200 nM of each dNTP, 1.5 mM MgCl_2 _and 1 U HotStar *Taq *(Qiagen) or Platinum *Taq *DNA polymerase (Invitrogen) in a 20 μl volume reaction. Thermocycling was as follows: 15 min 95°C, 30 cycles of 30 s 95°C, 1 min 55°C, and 1 min 72°C. Amplification products were resolved on silver stained 4% polyacrylamide gels [26]. Alternatively, DNA was digested with the blunt-end restriction enzymes *Alu*I, *Hae*III or *Rsa*I. Fragments were ligated to the GenomeWalker™ Adapter (Clontech) as recommended by the manufacturer and NBS5 and AP2 primers were used in PCR using the same conditions as described above.

In order to confirm that the amplification products were NBS encoding regions, fragments were excised from the gel, soaked in 100 μl of autoclaved deionized water overnight, and heated at 95°C for 5 min. A 5 μl aliquot was used as template for a PCR, under the same conditions as the original amplification. PCR products were sequenced on ABI automated DNA 377 or 3700 sequencers (Applied Biosystems). Sequences were processed and assembled using the Staden Package [27], with base calling performed by Phred [28]. Sequence similarities were identified using Blastx against local databases of predicted *Arabidopsis *proteins, *Arabidopsis *resistance genes, and Fabaceae proteins [29]. To aid in the translation of NBS encoding sequences, domains were searched using EstWise [30].

#### SNP marker development and genotyping

Twenty-four *Arachis *expressed sequence tag (EST) sequences of interest were selected for marker development. Fifteen of the ESTs are homologs of NBS encoding sequences, eight have diverse homologies and are responsive to late leaf spot or nematode inoculation ([20,31] and unpublished data), and one is a homolog of a dehydration responsive element (see Additional file 1). Primers were developed for the sequences using Primer3 . PCR products were resolved on 6% nondenaturing polyacrylamide gels and visualized by silver staining [26]. Amplification products that showed length polymorphism between the parents of the mapping population were used directly as markers. Size monomorphic products were sequenced and SNPs (single nucleotide polymorphisms) identified using the Staden Package software. SNP genotyping was performed using the SNaPshot™ single base extension method (Applied Biosystems).

### AFLP analysis

In order to increase the number of sequence characterized markers on the map and diversify the type of markers, AFLP was used [32]. PCR amplifications and electrophoresis were carried out as described above in NBS profiling (iii). Reactions were performed using *Pst*I and *Mse*I with 19 primer combinations. Primer information and sequences of mapped markers are provided in Aditional File 1.

### Linkage mapping

Linkage analysis was performed using all novel markers developed during this work plus the 204 microsatellites previously described [13]. The development of anchor markers and the analysis of synteny with other legumes is described elsewhere [15]. Segregation ratios of 1:2:1 or 3:1 of all segregating markers on the 93 F_2 _individuals were checked using a χ^2 ^test. The significance level was determined by using the false discovery rate (FDR) test at a level of 0.05 [33], which allows type-I error detection. The distortion types, if zygotic or gametic, were analyzed on the distorted markers using two χ^2 ^tests, according to [34]. Linkage analysis was done using Mapmaker Macintosh version 2.0 [35]. Linkage groups (LG) were established, using a minimum LOD score of 10.0 and a maximum recombination fraction (θ) of 0.35 with only the co-dominant, non-distorted markers [13]. The LOD score was then decreased to 3.0 in order to include new markers in the groups, using two-point analysis ("group" command). The new marker order within each LG was estimated by the matrix correlation method using the "first order" command. Alternatively, the exact position of new markers within each group was determined by using the "try" command, which compares the maximum-likelihood of each marker order after placing markers, one by one, into every interval of the established order. Marker orders were confirmed by permuting all adjacent triple orders ("ripple" command). Recombination fractions were converted into map distances in centimorgans (cM) using the Kosambi's mapping function.

### Bioassays

Phenotyping for resistance to *C. personatum *was performed on the parents of the mapping population, the F_1 _hybrid, individuals composing the mapping population itself, and *A. hypogaea *cv. IAC-Tatu-ST as susceptible control. The architecture of the wild derived diploid plants is not suitable for the application of standard field assays that are used on cultivated peanut, therefore a different approach was needed. Bioassays were done using detached leaves. This technique relies on the ability of peanut petioles to root into moistened cotton wool in a Petri-dish and thus remain alive for an extended period [36,37]. Plants were maintained over multiple years by pruning and when necessary making cuttings. An isolate of *C. personatum *collected from peanut in a field in Campinas (São Paulo State, Brazil) in the 2002/2003 season was maintained in Oat-agar medium. To avoid the isolate being attenuated, it was passaged through *A. hypogaea *leaves before use in bioassays. Leaves inoculated with fungal spores were maintained at 23–25°C and photoperiod of 10 h light and 14 h dark. Four replicates of each individual were analyzed 45 days after inoculation. Disease severity (susceptibility) was measured through the percentage of diseased leaf area (DLA). Statistical analyses were performed using Sigmastat (Jandel Scientific). Two bioassays were done, one in the 2003/2004 season and the other in the 2004/2005 season.

### QTL identification

Average diseased leaf area (DLA), in percentage, for the four replicates per individual in each bioassay was used for QTL mapping. The two bioassays were considered separately. QTLs were mapped by using the composite interval mapping method (CIM) [38,39] in the WinQTL Cartographer, version 2.5 [40]. CIM analysis was performed using Model 6, scanning intervals of 1 cM between markers and putative QTLs with a window size of 10 cM. The genetic effects and the gene action (dominance/additive effects) of significant QTL were obtained from multiple interval method (MIM) using all significant QTL from CIM [41]. Putative interactions between significant QTLs were analyzed using MIM. Graphic presentation of the LGs and QTLs was obtained by using MapChart, version 2.1 [42].

### Analysis of synteny

SSR markers in common between *Arachis *maps were considered as corresponding map points. Some SSR markers on this A-genome map were already in common with the SSR based map of cultivated peanut [14]. To increase the number of shared markers, selected SSRs placed in the cultivated map that had not been screened earlier for polymorphisms in the A-genome parents were screened and, when possible, genotyped and mapped in the A-genome.

The methodology for determining synteny of the A-genome map with *Lotus *and *Medicago *are described in detail elsewhere [15]. Briefly, all legume anchor markers [16-18,43] and most other markers mapped in the A-genome were sequence characterized. These sequences were used in BLAST as queries against the *Lotus *database from Kazusa DNA Research Institute (Japan), and against the pseudomolecules of *Medicago *using CViT blast (Chromosome Visualization Tool, ).

## Results

### Bioassays

**Figure 1:**
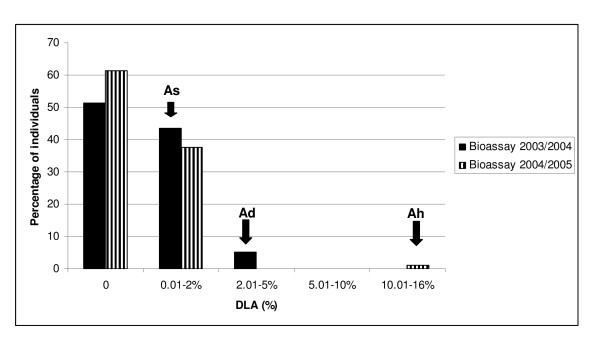
**Frequency distribution of disease symptoms in bioassays**. Frequency distribution of percentage of diseased leaf area (%DLA) in F_2 _lines derived from the cross between *A. stenosperma *V10309 (As) and *A. duranensis *K7988 (Ad), 45 days after infection with *C. personatum*. The susceptible control was *A. hypogaea *cv Tatu (Ah).

The observations of diseased leaf area did not follow a normal distribution within the population, being strongly biased towards resistance to the fungus. The susceptible parent *A. duranensis *K7988 had an average of 4.53% DLA (sd = 1.68), and differed significantly from the susceptible control *A. hypogaea *cv. Tatu, with 16.08% DLA (sd = 4.32), according to Tukey test, with P < 0.05. Seventy-three F_2 _plants had lower %DLA than the resistant parent *A. stenosperma *V10309 (0.15% DLA, sd = 0.00), of which 47 had no lesions (Figure 1).

### Development of markers and sequence analysis

#### Southern blot and SCAR markers

All nine RGA probes hybridized with both *A. stenosperma *V10309 and *A. duranensis *K7988, generating polymorphic markers. Four probe/enzyme combinations producing nine scorable markers were chosen for genotyping (S1_A_36/*Eco*RI; S1_A_37/*Eco*RI; S1S2_A_152/*Hind*III, and S4_A_164/*Hind*III). The SCAR markers were easy to score, but dominant.

#### NBS profiling

Initially a number of tests were done. Amplifications performed with common *Taq *polymerases (without hot start) produced a much larger number of fragments than with the hot start *Taq *polymerases. However, the consistency was lower and the polymorphic fragments when sequenced did not show similarity to any RGA from the Genbank. Tests were performed varying the number of selective bases at the end of the primers. As expected, the larger the number of selective bases, the fewer fragments were produced (on average 43, 26 and 12 for one, two or three selective bases, respectively; Additional file 2). Primers with two selective bases were mostly used, because they produced an apparently good combination of specificity, total number of fragments and number of polymorphic fragments.

Twenty-four primer combinations were chosen, amplifying 765 fragments, of which 138 were polymorphic and could be genotyped. Of these 138 fragments, 100 generated good quality sequences and 19 could be confirmed as being homologs to NBS containing genes. Some of the fragments were almost identical to RGAs previously isolated from *Arachis *[21]. The non-RGA fragments showed diverse homologies including for instance, kinases and an amylase (Aditional File 1). Some pairs of fragments were deduced by sequence and genotyping data to be co-dominant, and were scored as such.

#### SNP marker development and genotyping

Two of the 24 amplification products from ESTs were size polymorphic and SNPs were identified in all the other 22 sequences, with an average of one SNP per 210 bp. Single base extensions led to easy scorable, reliable co-dominant markers. Only one marker failed to genotype successfully.

#### AFLP

Standard AFLP using *Mse*I with the methylation sensitive *Pst*I was used to further enrich the map with sequence characterized markers. From the 19 primer combinations used, 144 fragments could be genotyped. Almost all of the mapped markers were sequence characterized and presented diverse homologies (see Additional file 1). Some pairs of fragments were deduced by sequence and genotyping data to be co-dominant, and were scored as such.

### Linkage map

**Figure 2:**
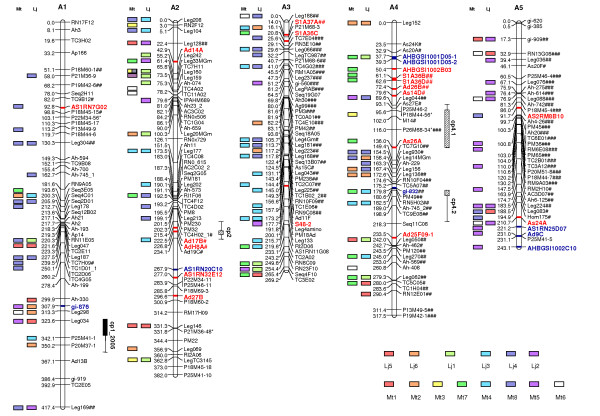
**A genetic linkage map of the A-genome of peanut – Linkage Groups A1 to A5**. A genetic linkage map, obtained through the analysis of 93 F_2 _plants, generated from a cross between two diploid wild A-genome *Arachis *species, *A. duranensis *× *A. stenosperma*. Segregation ratios deviating significantly from the expected ratios are indicated with # (P ≤ 0.05), ## (P ≤ 0.01) or ### for highly distorted markers. Numbers on the left of each group are Kosambi map distances. Markers that amplified two loci have numbers _1 and _2 after the marker name. Disease resistance candidates marker names that are homologs to *Arabidopsis *NBS encoding genes are highlighted in bold and red, other disease resistance candidate marker names are highlighted in bold and blue. QTLs are indicated as bars running alongside linkage groups. Marker correspondences with the chromosomes of the model legumes *Lotus japonicus *and *Medicago truncatula *are indicated as colored blocks.

**Figure 3:**
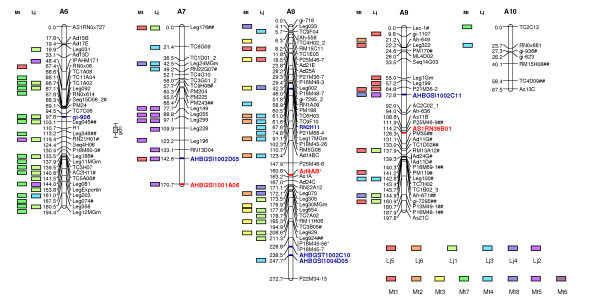
**A genetic linkage map of the A-genome of peanut – Linkage Groups A6 to A10**. A genetic linkage map, obtained through the analysis of 93 F_2 _plants, generated from a cross between two diploid wild A-genome *Arachis *species, *A. duranensis *× *A. stenosperma*. Segregation ratios deviating significantly from the expected ratios are indicated with # (P ≤ 0.05), ## (P ≤ 0.01) or ### for highly distorted markers. Numbers on the left of each group are Kosambi map distances. Markers that amplified two loci have numbers _1 and _2 after the marker name. Disease resistance candidates marker names that are homologs to *Arabidopsis *NBS encoding genes are highlighted in bold and red, other disease resistance candidate marker names are highlighted in bold and blue. QTLs are indicated as bars running alongside linkage groups. Marker correspondences with the chromosomes of the model legumes *Lotus japonicus *and *Medicago truncatula *are indicated as colored blocks.

Using a minimum LOD score of 3.0 and a maximum recombination fraction (θ) of 0.35, 369 markers mapped into 10 LGs. These markers included 188 microsatellites, 80 legume anchor markers, 46 AFLPs, 32 NBS profiling, 17 SNP, four RGA-RFLP and two SCAR markers. In total, 35 sequence confirmed candidate disease resistance genes were mapped, 21 being homologs to NBS-encoding genes and 14 homologs to other genes involved in plant defense, or genes induced by challenge with pathogens. LGs were numbered according to the first version of this map [13]. However, the inclusion of new markers resulted in 10 LGs instead of 11. The former LGs 8 and 11 joined, and were together named LG 8 (Figures 2 and 3).

A total of 142 (38.4%) out of the 369 mapped markers deviated from the expected F_2 _ratio of 1:2:1 (102 markers) or 3:1 (40 markers) at p < 0.05 level. A few distorted markers were found on seven of the 10 LGs (Figures 2 and 3). In contrast, LGs 3, 4 and 5 were basically composed of distorted markers. LGs 1, 2, 4 and 6 had markers with an excess of *A. stenosperma *alleles, while LGs 3, 5, and 9 were distorted toward the *A. duranensis *alleles. LGs 7, 8 and 10 showed distorted markers with an excess of heterozygotes.

### QTL identification

By using the permutation tests, the minimum LOD scores to declare as significant the putative QTL for resistance to *C. personatum *were estimated as 8.7 and 18.5 for the 2003/2004 and the 2004/2005 trials, respectively. The reason for these high values is the non-normal distribution of the phenotypic data, which is highly skewed towards resistance, and the presence of markers with distorted segregation in some regions of the genome (about 40% of distorted markers). Therefore, we have considered a QTL with LOD scores above 2.5 as significant, as suggested by [44].

Four QTLs were consistently identified in both bioassays, with LOD scores ranging form 9.9 to 17.3 (Table 1). These QTLs were mapped on LG 2 (cp2), LG 4 (cp4.1 and cp4.2), and LG 6 (cp6) (Table 1, Figure 2). The QTL cp2 showed the highest LOD in the position 226.7 cM with the closest marker being AdH8A, a homolog of NBS encoding disease resistance genes. The QTL cp4.1 was mapped within a cluster of candidate genes, while cp4.2 mapped between two candidate genes. QTL cp6 was located close to the anchor marker Leg346. An additional QTL was mapped only in the 2004/2005 trial, in LG 1 (cp1) close to the anchor marker Leg034.

**Table 1:** Quantitative trait loci for *Cercosporidium personatum *resistance identified by the multiple interval mapping (MIM) method.

**QTL**	**LG**	**Nearest marker(s)**	**Position** (cM)	**2003/2004 bioassay**				**2004/2005 bioassay**			
				
				LOD	a (%)	d (%)	d/a	LOD	a (%)	d (%)	d/a
**cp1**	**A1**	Leg034	328.4	-	-	-	-	11.8	4.2	1.7	0.40

**cp2**	**A2**	AdH8A*	226.7	10.7	9.7	2.1	0.22	11.9	12.1	5.2	0.43

**cp4.1**	**A4**	P25M46-2*/As26A*	106.4	14.1	22.6	7.6	0.34	12.7	14.2	4.5	0.32

**cp4.2**	**A4**	RN5H02/TC9E08	165.4	9.9	43.8	4.7	0.13	17.3	41.8	11.2	0.27

**cp6**	**A6**	Leg346	81.5	12.3	5.4	0.6	0.10	12.0	4.5	0.1	0.02

Legends: QTL = QTL name, LG = linkage group, LOD = maximum LOD score obtained for the QTL, (a) = additive and (d) = dominance effects (in percentage) and gene action obtained for the two bioassays.

*Markers AdH8A P25M46-2/As26A are RGAs

Five identified QTLs showed ratios of dominant to additive effects (*d/a*) less than 0.55, which might be interpreted as expression additive or only partially dominant [45]. For all the five identified QTLs, alleles from the resistant progenitor *A. stenosperma *increased resistance to late leaf spot. The QTL 4.2 showed the highest additive effect in the two trials, explaining almost half of the phenotypic variance observed. The other two QTLs detected close to RGA markers (cp2 and cp4.1) also showed significant additive and dominant effects in both trials. The QTL cp1, which was detected only in the 2004/2005 trial, and cp6 showed minor but significant additive effects (Table 1).

### Analysis of synteny

**Figure 4:**
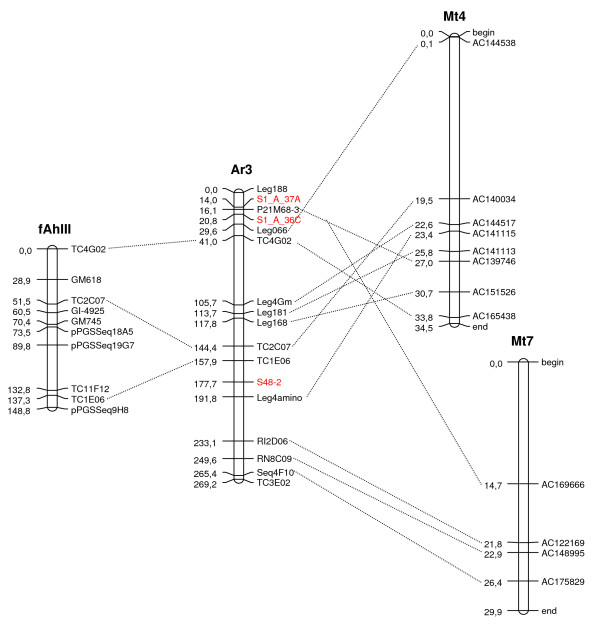
**An example of synteny between cultivated peanut, the A-genome *Arachis *map and *Medicago***. Synteny between a linkage group from cultivated peanut (fAhIII, meaning AhIII "flipped"), an A-genome linkage group (Ar3) developed here and two chromosomes of *Medicago *(Mt4 and Mt7). Synteny of the cultivated linkage group to *Medicago *can be inferred by using the A-genome map as a bridge, in addition the position of candidate genes on the map of cultivated peanut can be inferred. Marker names in *Medicago *are BAC clone identification codes. Genetic distances in *Arachis *are in cM, and in *Medicago *in Mbp of DNA. Candidate genes are highlighted in bold and red.

Both LGs 2 and 4 which contained mapped clusters of candidate genes and QTLs had poor or "shattered" synteny with the model legumes *Lotus *and *Medicago *(Figure 2). However, LG 3, on which mapped three candidate resistance genes, showed clear syntenies with *Lotus *and *Medicago*, and we chose this to illustrate how the diploid map can be integrated to the map of cultivated peanut and *Medicago *(Figure 4).

Using shared markers, alignments between LG3, LG6 and cultivated peanut linkage groups were possible. Therefore we chose to illustrate the integration of this diploid *Arachis *map, the map of cultivated peanut and *Medicago *using LG3 (Figure 4; for an alignment of LG6 see [14]).

## Discussion

Little is known about the genomic structure of *Arachis *and which regions control disease resistance. To the best of our knowledge in peanut, only markers linked to root-knot nematode resistance, resistance to the vector of groundnut rosette disease, rust and *Sclerotinia *blight have been published to date [46-50]. Markers linked to nematode resistance are integrated into a RFLP map, which is difficult to transfer to other populations, and the markers linked to aphid resistance are in an AFLP linkage map, which is sparse and difficult to transfer.

In this study we aimed to increase the information content of a previously published SSR-based *Arachis *map, begin to define the genomic regions that confer disease resistance and perhaps reveal major resistance gene clusters. For this we used two approaches: the mapping of candidate disease resistance genes, and the mapping of QTLs for resistance against one of the most important peanut diseases, late leaf spot.

For mapping candidate genes, we mainly focused on homologs of NBS domain encoding genes, and genes that respond to challenges with late leaf spot or nematodes ([1], unpublished data). We used four methods for marker development and genotyping, Southern blot, SCAR markers, NBS profiling and genotyping of SNPs using SNaPShot^®^. Although we were successful with all of these methods, we found marker development and genotyping with SNaPShot^® ^to be the most efficient, generating easy to score co-dominant markers. In total 35 sequence-confirmed candidate disease resistance genes were mapped, 21 being homologs to NBS-encoding genes.

For phenotyping we needed to use a method that was suitable for the distinct architecture of the wild diploids plants; standard field-based protocols for cultivated plants were not appropriate. Therefore, we used detached leaf bioassays [36], a method that measures one of the major components of late leaf spot resistance as defined for cultivated peanut. Plants were maintained for multiple years by pruning, transplanting, and by taking cuttings if necessary, this allowed the performance of bioassays on the same population in different years.

For QTL analysis we used CIM and MIM methods. Although these methods are designed for data where phenotypic variation is normally distributed, they work with non-normal distributed traits [51-55]. Of the QTLs identified, four of the five QTLs were consistent between bioassays done in different years. All QTLs had LOD scores above 9.9, well above the 2.5 limit suggested for significance by [44]. In one of the trials (2003/2004), LOD scores exceeded the minimum threshold calculated by permutation – a method that is known to overestimate significant scores for non-normal data. Therefore, the support for the QTLs is good, though clearly, the aim of bioassays was not to identify QTLs that could immediately be used with confidence in cultivated peanut. Rather the aim was to give indications of what parts of the *Arachis *genome are involved in disease resistance, and to consider these results together with the map positions of candidate genes.

The comparison of RGA map positions and QTLs is striking. The markers closest linked to two of the five QTLs were RGAs. This strongly suggests the involvement of NBS encoding genes in the resistance response. The best known cases of NBS encoding disease resistance genes are monogenic and dominant. However, in this study the resistance seems to be polygenic and possibly partially dominant. These results are broadly consistent with previous data on the inheritance of late leaf spot resistance in cultivated peanut (reviewed by [3]). The sum of the genetic effects of the QTLs calculated using MIM was close to 100% in both trials. Although these effects are probably overestimated, they provide a good comparison between the genetic effects of each QTL and the major QTLs could be identified. For the two trials, the QTL cp4.2 showed additive effects that explained almost half of the total phenotypic variance (Table 1). This QTL was located between the microsatellite markers RN5H02 and TC9E08 (Figure 2), close to a QTL for seed-weight (data not shown). In consequence, after validation in other mapping populations, it is a good candidate for MAS. Two additional QTLs (cp2 and cp4.1) showed considerable additive effects that explained, together, ~30% of the variance. Both QTLs were located close to RGA markers (AdH8A and As26A, respectively). The upper portion of LG 4, where this QTL was mapped is RGA-rich (Figure 2). Many authors have reported close associations between RGAs and disease resistance loci and QTL (e.g., [40,56-58]). Therefore, such RGAs can also be useful for MAS of resistant genotypes. Recombinant inbred lines generated from a tetraploid population {*A. hypogaea *× (*A. ipaënsis *× *A. duranensis*)^4×^} are being phenotyped for resistance/susceptibility to late leaf spot, aiming at the validation of the results obtained here.

The best characterized legume genomes are those of the model plants *Lotus *and *Medicago*, which thus serve as useful references for comparison with *Arachis*. The *Medicago *genome harbors two "super-clusters" of resistance gene analogs, one in the upper region of chromosome 3 and one in the lower region of chromosome 6; clusters are also present in the upper regions of chromosomes 4 and 8 [59]. In *Lotus*, clusters of resistance gene analogs are present on chromosomes 1, 2 and 3 [60]. Interestingly, synteny between *Medicago *and *Lotus *appears to be poor in many of the genomic regions that harbor major resistance gene clusters [59-61]. Therefore, it is notable that *Arachis *A-genome LGs 2 and 4, which harbor the most prominent clusters of candidate genes and QTLs, showed shattered synteny with both *Lotus *and *Medicago*. It is possible that the breakage of synteny in resistance gene clusters may be due to their fast evolving nature, and their clustering with another fast evolving component of the genome, retrotransposons [15]. However, not all candidate disease resistance genes containing regions of this A-genome map have poor synteny, and an example of the integration of LG III of cultivated peanut, LG 3 of the A-genome map and *Medicago *chromosomes is shown in Figure 4. The ability to integrate different maps in this way will increase with future work and increased marker densities.

## Conclusion

The present study mapped 35 candidate genes and five QTLs for late leaf spot disease resistance. The study indicated several regions within the *Arachis *genome as being involved in controlling disease resistance. In particular, clustering of the candidate genes and QTLs suggests that the upper region of LG 4 and the lower region of LG 2 are likely to control disease resistance and to harbor clusters of disease resistance genes in *Arachis*.

## Authors' contributions

SCMLB was the main author responsible for the writing of the manuscript, doing the bioassays, participated in marker development, analysis of data and the co-ordination of the study. ACVFJ and DMTAF were the main authors responsible for developing and genotyping candidate genes. PMG, SN, BV, RWP, JP, and MP also participated in marker development and genotyping. MCM participated in genotyping and did the linkage and QTL analysis. APF participated in bioassays. RV participated in the marker screening and genotyping work that enabled the linkage of *Arachis *maps. DJB participated in writing the manuscript, marker development, analysis of data and co-ordination of the study. All authors read and approved the final manuscript.

## Supplementary Material

Additional file 1 **Marker homologies and information**. The file provides information on all markers used in this work: sequence homologies, position in linkage groups, primer sequence and reference. Click here for file

Additional file 2 **Patterns of DNA bands amplified by NBS profiling**. The file is a supplementary figure showing patterns of DNA bands amplified by NBS profiling with different numbers of selective bases resolved on polyacrylamide gel. Click here for file
