# Supplementary material for: Identification of candidate genome regions controlling disease resistance in Arachis
Source: BMC Plant Biol. 2009 Aug 22;9:112. doi: 10.1186/1471-2229-9-112 (PMC2739205; doi:10.1186/1471-2229-9-112)
Supplement: Additional file 2 — Patterns of DNA bands amplified by NBS profiling. The file is a supplementary figure showing patterns of DNA bands amplified by NBS profiling with different numbers of selective bases resolved on polyacrylamide gel. [file 1471-2229-9-112-S2.doc]

**Additional file 2:** Patterns of DNA bands amplified by NBS profiling with different numbers of selective bases resolved on polyacrylamide gel. V is *A. stenosperma* V10309, K is *A. duranensis* K7988, and H is the hybrid F_1_ plant. Less bands are amplifed with more selective bases.

**
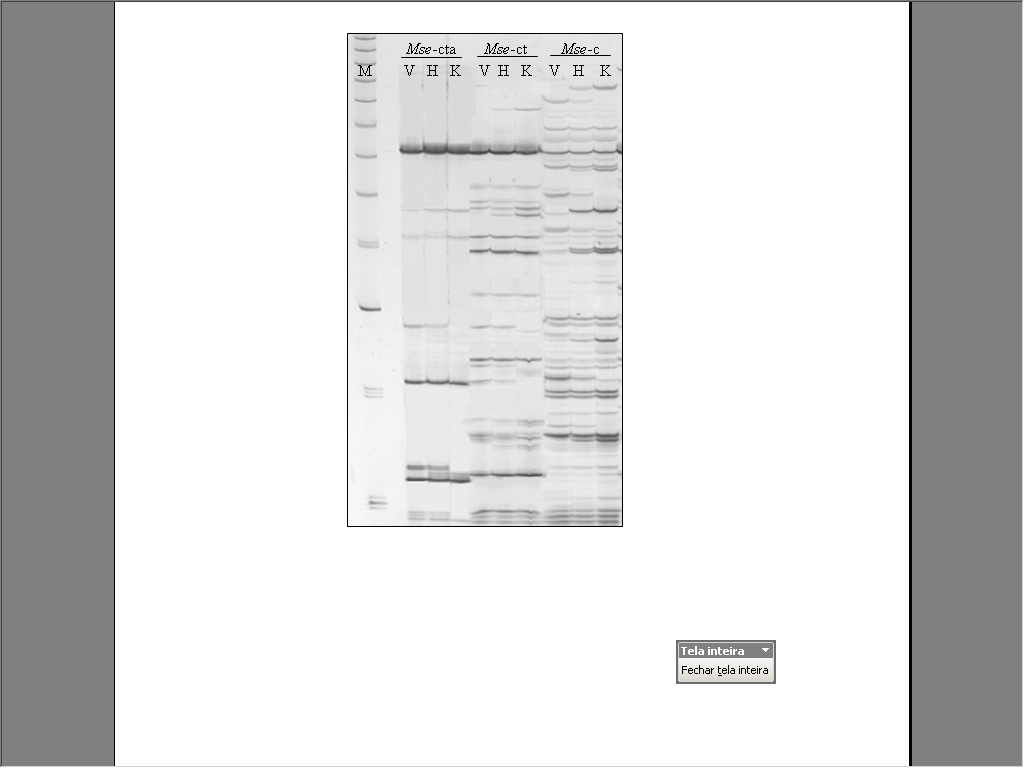
**
